# DeepRetroMoCo: deep neural network-based retrospective motion correction algorithm for spinal cord functional MRI

**DOI:** 10.3389/fpsyt.2024.1323109

**Published:** 2024-06-28

**Authors:** Mahdi Mobarak-Abadi, Ahmad Mahmoudi-Aznaveh, Hamed Dehghani, Mojtaba Zarei, Shahabeddin Vahdat, Julien Doyon, Ali Khatibi

**Affiliations:** ^1^ Institute of Medical Science and Technology, Shahid Beheshti University, Tehran, Iran; ^2^ Cyberspace Research Institute, Shahid Beheshti University, Tehran, Iran; ^3^ Neuro Imaging and Analysis Group (NIAG), Research Center for Molecular and Cellular Imaging (RCMCI), Tehran University of Medical Sciences, Tehran, Iran; ^4^ Department of Applied Physiology and Kinesiology (DAPK), University of Florida, Gainesville, FL, United States; ^5^ Montreal Neurological Institute, McGill University, Montreal, QC, Canada; ^6^ Centre of Precision Rehabilitation for Spinal Pain, School of Sports Exercise and Rehabilitation Sciences, University of Birmingham, Birmingham, United Kingdom; ^7^ Centre for Human Brain Health, University of Birmingham, Birmingham, United Kingdom

**Keywords:** fMRI, spinal cord, motion correction, deep learning, unsupervised

## Abstract

**Background and purpose:**

There are distinct challenges in the preprocessing of spinal cord fMRI data, particularly concerning the mitigation of voluntary or involuntary movement artifacts during image acquisition. Despite the notable progress in data processing techniques for movement detection and correction, applying motion correction algorithms developed for the brain cortex to the brainstem and spinal cord remains a challenging endeavor.

**Methods:**

In this study, we employed a deep learning-based convolutional neural network (CNN) named DeepRetroMoCo, trained using an unsupervised learning algorithm. Our goal was to detect and rectify motion artifacts in axial T2*-weighted spinal cord data. The training dataset consisted of spinal cord fMRI data from 27 participants, comprising 135 runs for training and 81 runs for testing.

**Results:**

To evaluate the efficacy of DeepRetroMoCo, we compared its performance against the sct_fmri_moco method implemented in the spinal cord toolbox. We assessed the motion-corrected images using two metrics: the average temporal signal-to-noise ratio (tSNR) and Delta Variation Signal (DVARS) for both raw and motion-corrected data. Notably, the average tSNR in the cervical cord was significantly higher when DeepRetroMoCo was utilized for motion correction, compared to the sct_fmri_moco method. Additionally, the average DVARS values were lower in images corrected by DeepRetroMoCo, indicating a superior reduction in motion artifacts. Moreover, DeepRetroMoCo exhibited a significantly shorter processing time compared to sct_fmri_moco.

**Conclusion:**

Our findings strongly support the notion that DeepRetroMoCo represents a substantial improvement in motion correction procedures for fMRI data acquired from the cervical spinal cord. This novel deep learning-based approach showcases enhanced performance, offering a promising solution to address the challenges posed by motion artifacts in spinal cord fMRI data.

## 1 Introduction

Spinal cord functional magnetic resonance imaging (fMRI) has become increasingly popular for exploring intrinsic neural networks and their role in pain modulation, motor learning, and sexual arousal (1, 2). There are unique challenges in data acquisition and preprocessing, such as relatively small cross-sectional dimension, the variable articulated structure of the spine between individuals, low signal intensity in standard gradient-echo echo-planar T2^*^-weighted fMRI, and voluntary (bulk motion) or involuntary (fluctuation of cerebrospinal fluid due to respiration and heartbeat) movements during image acquisition (3–5). Spinal cord motions can cause signal alterations across volumes, which decrease the temporal stability of the signal and ultimately increase false-positive and -negative discovery rates (6–8).

Despite advances in fMRI motion correction, there are problems in extrapolating the motion correction algorithm developments in the brain to the brainstem and spinal cord. In brain fMRI, we generally utilize six degrees of freedom rigid-body registration of a single volume to a reference, which can be a preselected volume or an average volume (9, 10). This method is non-robust and insufficient for spinal cord fMRI preprocessing due to the non-rigid motion of the spinal column and physiological motion from swallowing and the respiratory cycle (3, 11). Along with the release of the Spinal Cord Toolbox (SCT), sct_fmri_moco was introduced for motion correction in the spinal cord (12). The basis of sct_fmri_moco is slice-by-slice regularized registration for spinal cord algorithm (SliceReg) that estimates slice-by-slice translations of axial slices while ensuring regularization constraints along the *z*-axis (13).

In the past few years, we have seen an interest in the application of artificial intelligence in medical image processing (14–16). In spinal cord imaging, deep learning has been used for the segmentation of the spinal cord and CSF in structural T1- and T2-weighted images. DeepSeg as a fully automated framework based on convolutional neural networks (CNNs) is proposed to apply spinal cord morphometry for segmenting the spinal cord, as part of SCT (17–19). More recently, the K-means clustering algorithm has been employed specifically for delineating segments of the spinal cord within the thoracolumbar region, demonstrating its utility in identifying distinct anatomical structures within this complex area (20) This application is particularly notable for its ability to differentiate between the spinal cord and surrounding tissues, offering a promising automated approach for spinal cord morphometry. A robust and automated CNN model with two temporal convolutional layers is introduced for motion correction in brain fMRI, and the following regression employs derived motion regressors (21).

Studies in the field of registration are generally divided into two categories: learning-based and non-learning based. In the non-learning category, extensive work has been done in the field of 3D medical image registration (22–27). Some models are based on optimizing the field space of displacement vectors, which include elastic models (22, 28), statistical parametric mapping (29), free-form deformations with b-spline (29), and demons (23). Common formulations include Large Diffeomorphic Distance Metric Mapping (LDDMM) (30, 31), DARTEL (24), and standard symmetric normalization (SyN) (25). There are several recent articles in learning-based studies that have suggested neural networks for registering medical images, and most of them require ground truth data or any additional information such as segmentation results (32–35).

To the best of our knowledge, no prior study utilized AI for motion correction in the spinal cord fMRI. This study aimed to train a deep learning-based CNN via unsupervised learning to detect and correct motions in axial T2*-weighted spinal cord data. We hypothesize that our method can improve the outcome of motion correction and reduces the preprocessing time as compared to the existing methods.

## 2 Methods

### 2.1 Fixing centerline as preprocessing

In our preprocessing approach, data alignment in each slice over time was conducted using a centerline within the spinal cord, extracted using the spinal cord toolbox. To adjust for points outside the expected range or missing, we used third-degree b-spline interpolation and the interquartile range method to determine the centerline coordinates’ boundaries. This interpolation not only corrects for misalignments but also preserves the natural curvature of the spinal cord in three-dimensional space, maintaining the anatomical fidelity of the neck—a critical aspect when considering the complex geometry of the spinal cord.

In our pursuit of optimizing efficiency and effectiveness, we meticulously evaluated computational costs, particularly during the initial centerline realignment stage. This evaluation focused on correcting displacements along two axes: the *x*-axis, corresponding to lateral shoulder movements, and the *y*-axis, associated with vertical chest movements due to breathing.

Our comprehensive analysis revealed a notable finding: *y*-axis corrections were significantly more effective, a result that was anticipated given the constant position of shoulders during scans. Our numerical analysis underscored this, showing a higher variance in the *y*-direction (1.1) compared to the *x*-direction (0.52), indicating a more pronounced scattering in the *y*-direction and underscoring the predominance of chest movements. Consequently, *y*-axis corrections alone captured the essential adjustments required in our dataset and model architecture during the centerline realignment phase.

Adjustments for *x*-axis movements were addressed in subsequent stages for full spatial transformation. However, integrating both *x* and *y* corrections at the initial stage did not markedly improve outcomes over *y*-axis corrections alone (**Table 1**) but led to increased computational costs and extended processing time by approximately 45%.

**Table 1:** Impact of Y-only vs. X and Y centerline correction on tSNR and DVARS.

Methods		Spinal cord	CSF	Mean (S)	
		Mean (SD)			
**tSNR**	Post-correction *Y* only	7.1 (2.41)	4.03 (1.17)	**DVARS**	0.03 (0.010)
	Post-correction *X* and *Y*	6.9 (1.9)	4.13 (1.31)		0.03 (0.009)

**Figure 1:**
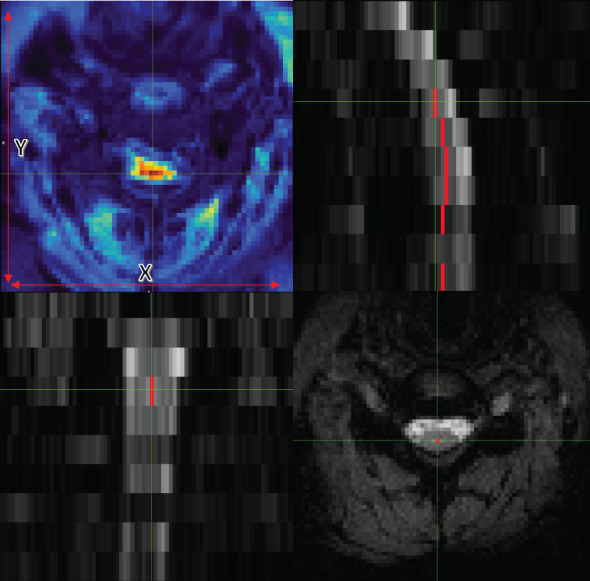
Axial (bottom right), coronal (bottom left), and sagittal (top right) views of data with the centerline. The image on the top left also shows tSNR with the x and y guidelines.

Given these insights, we strategically focused on *y*-axis corrections for centerline realignment, aiming for an optimal balance between model performance and operational efficiency. This approach streamlined our procedures and reduced unnecessary computational expenditure, emphasizing our commitment to refining and improving our methodologies with a focus on cost efficiency and effectiveness (see **Figure 1**).

However, given that literature and empirical observations suggest minimal movement in the *z*-direction in spinal cord fMRI (36), we did not perform corrections in the *z*-direction. This targeted approach in preprocessing was designed to enhance the training efficiency and accuracy of our DeepRetroMoCo algorithm. Notably, the final output of DeepRetroMoCo’s spatial transformation map offers full freedom for correction across all considered directions, ensuring a comprehensive and effective motion correction for the entire dataset.

### 2.2 Unsupervised deep learning network architecture

#### 2.2.1 Convolutional neural network architecture

**Figure 2:**
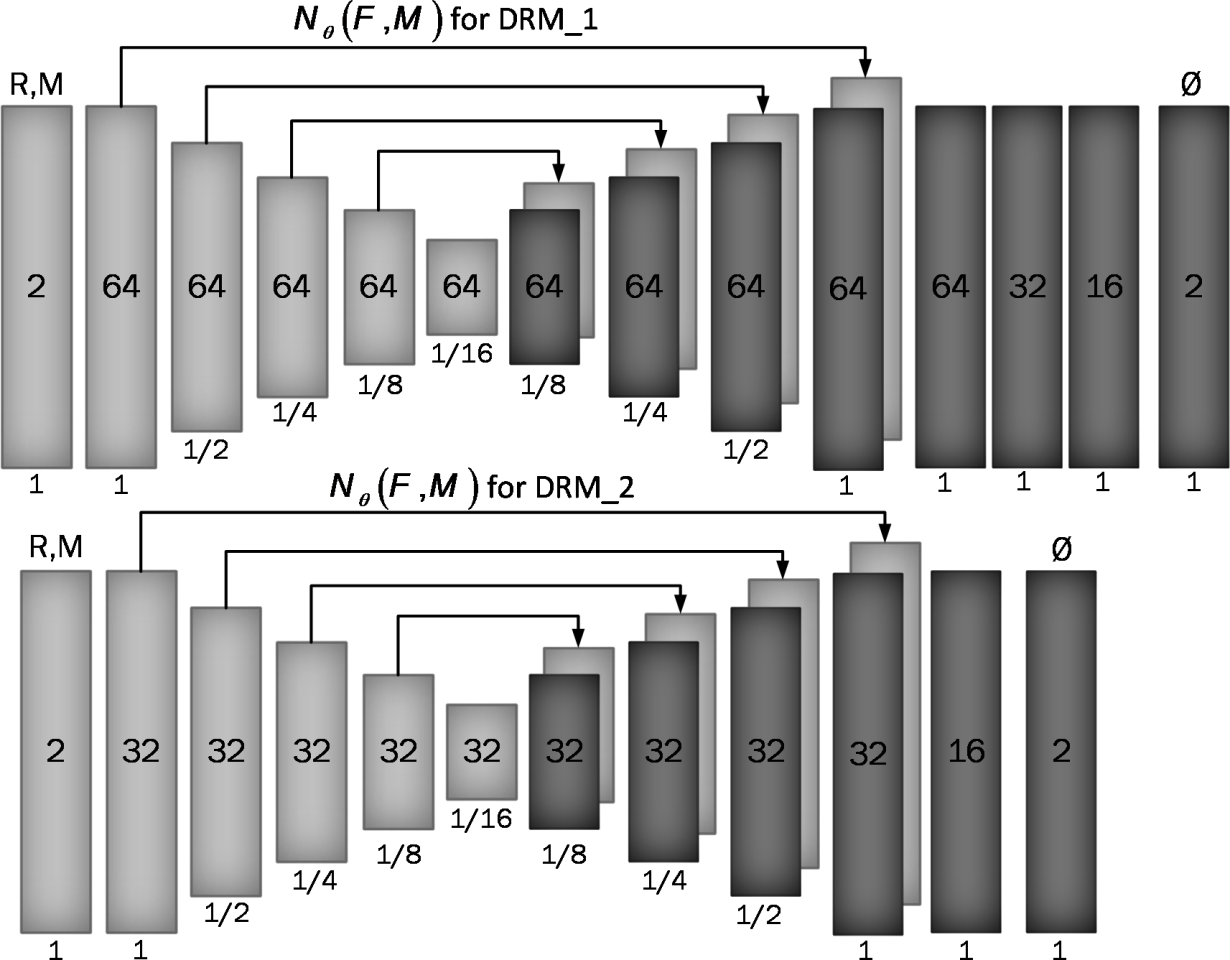
Proposed convolutional architectures implementing g_θ (F,M). Each rectangle shows a 2D volume in which two fixed and moving images are connected. The number of channels inside each rectangle is shown and the spatial resolution is printed below it according to the input volume. The first model has a larger architecture and more channels than the second model.

**Figure 3:**
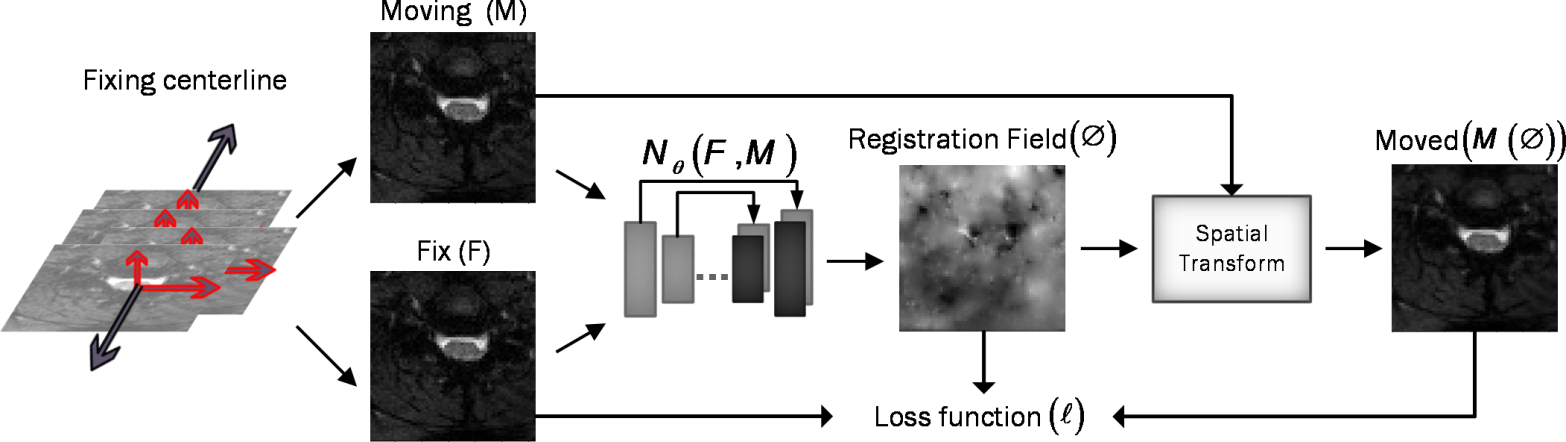
Overview of DeepRetroMoco. As a preprocess, we align the data in two dimensions based on the centerline, and then we register the moving image (M) to the fixed image (F) by learning function parameters (N). During training, an ST was used to warp the moving image with the registration field, and in this operation, the loss function compares *M*(Ø) and F using the smoothness of Ø.

Assume *M* and *F* are two images of the same slice defined in the *N*-dimensional spatial domain Ω ⊂ *R^N^*. We are focusing on *N* = 2 because the type of data we are using is “functional,” containing single-channel grayscale data. Additionally, our network focuses on the Axial view. The fixed image *F* is the reference volume, so it can be the first, middle, average, or any of the volumes, and *M* is the rest of the time-series images. Before training the network, we align *F* and *M* using our fixing Centerline method, which we describe in the following section, so that the only misalignment between the volumes is nonlinear. We then use a CNN structure similar to UNet (37, 38) to model a *N_θ_* (*F,M*) = Ø function, which includes an encoder and decoder with skip connection (**Figure 2**): where ∅ is the register map between the two input images and the θ learned parameter of the network. In this map, for each voxel *p* ∈ Ω, there is a position where *F*(*p*) and the warped image M(∅(p)) have the same anatomical position. Therefore, our network takes the concatenated images *F* and *M* as input and calculates the registration flow field based on θ . In the next step, it uses the spatial transformation operator to warp the moving image based on the flow field and evaluates the similarity between *M* and *F* and θ update. **Figure 3** shows our introduced architecture and an integrated input by concatenating *F* and *M* in two channels of the 2D image.

In both the encoder and decoder stages, we use two-dimensional convolution with a 3×3 kernel size and leaky Relu activation. The hierarchical properties of the concatenated image pair are captured by the convolution layer, which is required to estimate ∅ . We also use stride convolution to decrease the spatial dimensions and get to the smallest layer. During the encoding steps, features are extracted by downsampling, and during the decoding and upsampling steps, the network propagates the trained features from the previous step directly to the layer that generates the registry by using a skip connection. A decoder’s output size  (∅) is equal to the input image *M*.

We used two architectures to examine a trade-off between speed and accuracy. These two structures, DRM_1 and DRM_2, differ in their architectural complexity at the end of the decoder. DRM_1, being the more complex model, uses additional layers at the end of the decoder and more channels throughout, resulting in a total of 467,474 parameters. In contrast, DRM_2 is designed with fewer parameters, totaling 116,370, making it a more compact model. This difference in the number of parameters reflects the variations in computational complexity and capacity between the two models.

To find the optimal theta parameter, we used the stochastic gradient descent method to minimize the loss function ℒ : (Equation 1)


(1) $$\hat{\theta }=arg\underset{\theta }{min}\left[E_{\left(F,M\right)∼D}\left[ℒ\left(F,M,g_{\theta }\left(F,M\right)\right)\right]\right]$$

where *D* is the empirical distribution. It should be noted that we do not need supervisor information such as Atlas or T1 images.

The ${ℒ}_{Unsupervised}$ consists of two parts: (Equation 2) ${ℒ}_{sim}$ , which measures the similarity between *F* and M(∅) , and $\;{ℒ}_{reg}$ , which measures the smoothness of the registration field. Thus, our total loss function is as follows:


(2) $$ℒ\left(F,M,\emptyset \right)={ℒ}_{sim\;}\left(F,M\left(\emptyset \right)\right)+\lambda {ℒ}_{reg\;}\left(\emptyset \right)$$

λ is the regulation parameter.

We used two different cost functions for ${ℒ}_{sim}$ : mean square error (Equation 3) and normalize cross-correlation (Equation 4), which is a common metric due to robust intensity variations. The first cost function, the mean square error, is as follows:


(3) $$MSE=\frac{1}{{\left(Image\;sigma\right)}^{2}}\times \frac{1}{N}\times \sum_{p_{i}\in \Omega }{\left(F\left(p_{i}\right)-M\left(\emptyset \left(p_{i}\right)\right)\right)}^{2}$$

Here, $p_{i}$ is the position of the pixels and Image sigma is equal to 1 in this work. In addition, the fact that MSE is close to 0 indicates better alignment. The second cost function, normalizing cross-correlation, is as follows:


(4) $$CC={\;}_{\sum }^{p\in \Omega }\frac{{\left(\sum_{p_{i}}\left(F\left(p_{i}\right)-\hat{F}\left(p\right)\right)\left(M\left(\emptyset \left(p_{i}\right)\right)-\hat{M}\left(\emptyset \left(p\right)\right)\right)\right)}^{2}}{\left(\sum_{p_{i}}\left(F\left(p_{i}\right)-\hat{F}\left(p\right)\right)\right)\left(\sum_{p_{i}}\left(M\left(\emptyset \left(p_{i}\right)\right)-\hat{M}\left(\emptyset \left(p\right)\right)\right)\right)}$$

Let $F\left(p_{i}\right)$ and $M\left(\emptyset \left(p_{i}\right)\right)$ be the image intensities of fixed and moving images, respectively, and $\hat{F}\left(p\right)$ and $\hat{M}\left(\emptyset \left(p\right)\right)$ be the local mean at position *p*, respectively. The local mean is computed over a local $n^{2}$ window centered at each position *p* with *n* = 3 in this work.

By minimizing ${ℒ}_{sim}$ , we seek to approximate M(∅(p)) from F(p) , but it may cause a discontinuity in ∅ , so we used spatial gradients to regulate the deformation field between the voxel’s neighborhood, as follows: (Equation 5)


(5) $${ℒ}_{\text{regularization }}=\sum_{\text{p}\in \text{Ω}}{\left\|\nabla \emptyset \left(\text{p}\right)\right\|}^{2}=\sum {\left\|\frac{\partial \emptyset }{\partial \text{x}}\right\|}^{2}+{\left\|\frac{\partial \emptyset }{\partial \text{y}}\right\|}^{2}+{\left\|\frac{\partial \emptyset }{\partial \text{z}}\right\|}^{2}\;\;$$

This cost function is applied to the network’s output vectors and controls the size of the vectors by deriving the vectors in each direction.

#### 2.2.2 Spatial transformation function

Our spatial transformation function (STF) is critical for learning the transformation parameters θ, which align the moving image (M) with the fixed image (F) by minimizing their dissimilarity (39). This process is distinct from Pix2pix’s approach, which typically relies on paired examples in a supervised learning context for image-to-image translation. Our unsupervised method, instead, leverages the inherent structure within the data, learning θ directly from the alignment of M and F without the need for such pairs.

The STF generates a sampling grid using the predicted transformation parameters θ, creating a deformed version of M [notated as M(∅)]. It is worth noting that the STF in our network learns this deformation field in an unsupervised manner, which is not directly comparable to the Pix2pix model that requires paired training data. Moreover, our method uses bilinear interpolation at non-integer positions to ensure a smooth and continuous transformed image, which is critical for maintaining anatomical structure after transformation.

To further distinguish our work from Pix2pix, we use a unique loss function that balances the similarity between F and the warped image M(∅) with the regularization of the deformation field to ensure smoothness. This loss function is key for our network to produce a deformation field that enables precise alignment while preserving the structural integrity of the images.

### 2.3 Experiments

#### 2.3.1 Dataset

The data used for this experiment include 30 subjects with T2*-weighted MRI scans acquired from a 3T TIM Trio Siemens scanner (Siemens Healthcare, Erlangen, Germany) equipped with a 32-channel head coil, and a 4-channel neck coil was used for the imaging to investigate the functional activity in the brain and the spinal cord (40). All subjects were scanned twice. Five runs were collected in the first session and three runs were collected in the second session. Sessions were acquired 1 week apart. This resulted in 240 runs. We only used the data from the neck coil and cervical spinal cord in this study.

The dataset included 8–10 slices that covered the cervical spinal cord from C3 to T1 spinal segmental levels and were oriented parallel to the spinal cord at the C6 level. The FoV of the slices was $132\times 132\;{\text{mm}}^{2}$ , with voxel sizes of $1.2\times 1.2\times 5\;{\text{mm}}^{3}$ and a 4-mm gap between them. The flip angle was 90°, and the bandwidth per pixel was 1,263 Hz, resulting in an echo spacing of 0.90 ms. 7/8 partial Fourier and parallel imaging (*R* = 2, 48 reference lines) was utilized again, resulting in a 43.3-ms echo train length and a 33-ms echo time. Finally, the TR for all slices was 3,140 ms, except for three subjects, who had TRs of 3,050 ms or 3,200 ms (depending on each participant’s coverage within the field of view). In the data preprocessing phase, we removed any instances of data that were deemed to be of low quality or exhibited discrepancies in data points when benchmarked against other datasets. Consequently, we curated a dataset comprising 27 subjects across 216 functional runs, of which 135 were allocated for the training set and the remaining 81 were allocated for the testing set. The training dataset was further partitioned into a 70:30 split for model training and validation, respectively. The validation subset played a crucial role in both the selection and performance evaluation of our proposed deep-learning models.

#### 2.3.2 Evaluation

Since there is no gold standard for direct evaluation of functional registration or motion correction performance, we used two functional measures to check the signal strength of each subject or to examine signal variations in the group of volumes after predicting them by the network.

##### 2.3.2.1 Temporal signal-to-noise ratio

Temporal signal-to-noise ratio (tSNR) is used to quantify the stability of the BOLD signal time series and is calculated by dividing the mean signal by the standard deviation of the signal over time (Equation 6).


(6) $$tSNR=\frac{\bar{S}}{\sigma_{t\_noise}}\;\;\;\;\;\;\;\;\;\;\;\;\;\;\;\;\;\;\;\;\;\;\;\;\;\;\;\;\;$$

where $\bar{S}$ is the mean signal over time and σ is the standard deviation across time. A better motion correction algorithm will result in greater tSNR values by reducing signal variations in the BOLD time series due to motion.

##### 2.3.2.2 DVARS

DVARS (D, temporal derivative of time courses, VARS, variance over voxels) shows the signal rate changes in each fMRI data frame. In an ideal data series, its value depends on the temporal standard deviation and temporal autocorrelation of the data (41) and calculates the changes in the values of each voxel at each time point compared to its previous time point (42). DVARS was calculated in the whole image to find a metric that demonstrated the standard deviation of temporal difference images in the 4D raw data (43). DVARS was calculated using the following equation: (Equation 7)


(7) $$DVARS{\left(\Delta I\right)}_{i}=\sqrt{{\left(\Delta I_{i}\left(x\right)\right)}^{2}}=\sqrt{{\left(I_{i}\left(x\right)-I_{i-1}\left(x\right)\right)}^{2}\;}$$

In this equation, $\Delta {\text{I}}_{i}\left(x\right)\;$ is used as local image intensity on the frame. DVARS could result in more accurate modeling of the temporal correlation and standardization because it is obtained by the most short-scale changes (41). The best value for this parameter is zero, and the closer it is to zero, the better the result.

We extracted the tSNR and DVARS parameters of output results by using the SCT toolbox and the FSL toolbox (44). For more accurate analysis of the tSNR parameter, we manually segmented the data into two parts, spinal cord and CSF, using the FSLeyes toolbox. Analyses compared the outcome of SCT and our method (DeepRetroMoco).

#### 2.3.3 Statistical analysis

All statistical analyses were carried out using IBM SPSS Statistics (V. 25 IBM Corp., Armonk, NY, USA) with α< 0.05 as the statistical significance threshold. The Kolmogorov–Smirnov test was used to determine the normality of the parameters. For statistically significant results, the mean of normal data for each method was processed using one-way ANOVA with repeated measures in within-subjects comparison, followed by a multiple comparison *post-hoc* test with Bonferroni correction.

#### 2.3.4 Implementation

In the course of our experiment, we evaluated our deep learning network’s performance both with and without the application of the “Fixing Centerline” preprocessing step. Our network underwent training over 200 epochs, each consisting of 150 iterations. The training process was executed using the Keras library with a TensorFlow backend (45) on an NVIDIA GEFORCE RTX 1080 GPU, which, on average, took 23 h to complete a full training cycle. To enhance our efficiency, we utilized the high-powered computational environment of Google Colab for model assessment and hyperparameter tuning, resulting in a more expedited analysis and learning process.

The optimization parameter we used was Adam, with a learning rate of $1e^{-4}$ (46). We trained our two models, the simpler DRM_2 and the more complex DRM_1, using two different cost functions, namely, normalized cross-correlation (NCC) and mean squared error (MSE), each with varying lambda values until convergence. Batch normalization was implemented to stabilize the training process, and min–max normalization was used during preprocessing to normalize the input data.

In our study, we designed an optimized data generator to deliver fMRI data to the network efficiently. This data generator operates by randomly selecting subjects and slices, ensuring that the training and validation sets are disjoint at the subject level. It then chooses a pair (fixed and moving images) from the corresponding volume, adhering to the specified batch size of 100 images. This approach of random selection at the subject level allows for the assessment of the model’s performance on new, unseen data, providing a robust evaluation of its noise-correction capabilities in a real-world setting where each subject’s data presents unique variations.

In comparison between the models, we chose the model that has better results in terms of our desired metric (tSNR) on the validation data. Then, we select one of the cost functions. Our code and model parameters are available online at https://github.com/mahdimplus/DeepRetroMoco.

## 3 Results

### 3.1 Model selection

**Table 1** displays the average of our method’s tSNR values in the validation data utilizing two distinct cost functions. The first model, DRM 1, outperforms DRM 2 in both Losses MSE and NCC by a slight margin. Furthermore, when the validation data of two cost functions in the first model are examined, NCC with an average of 10.13 ± 1 has better outcomes for the motion correction target based on the tSNR and statistical analysis, *t*(39) = 2.63, *p*< 0.05.

### 3.2 Visual comparison of motion correction protocols

**Figure 4:**
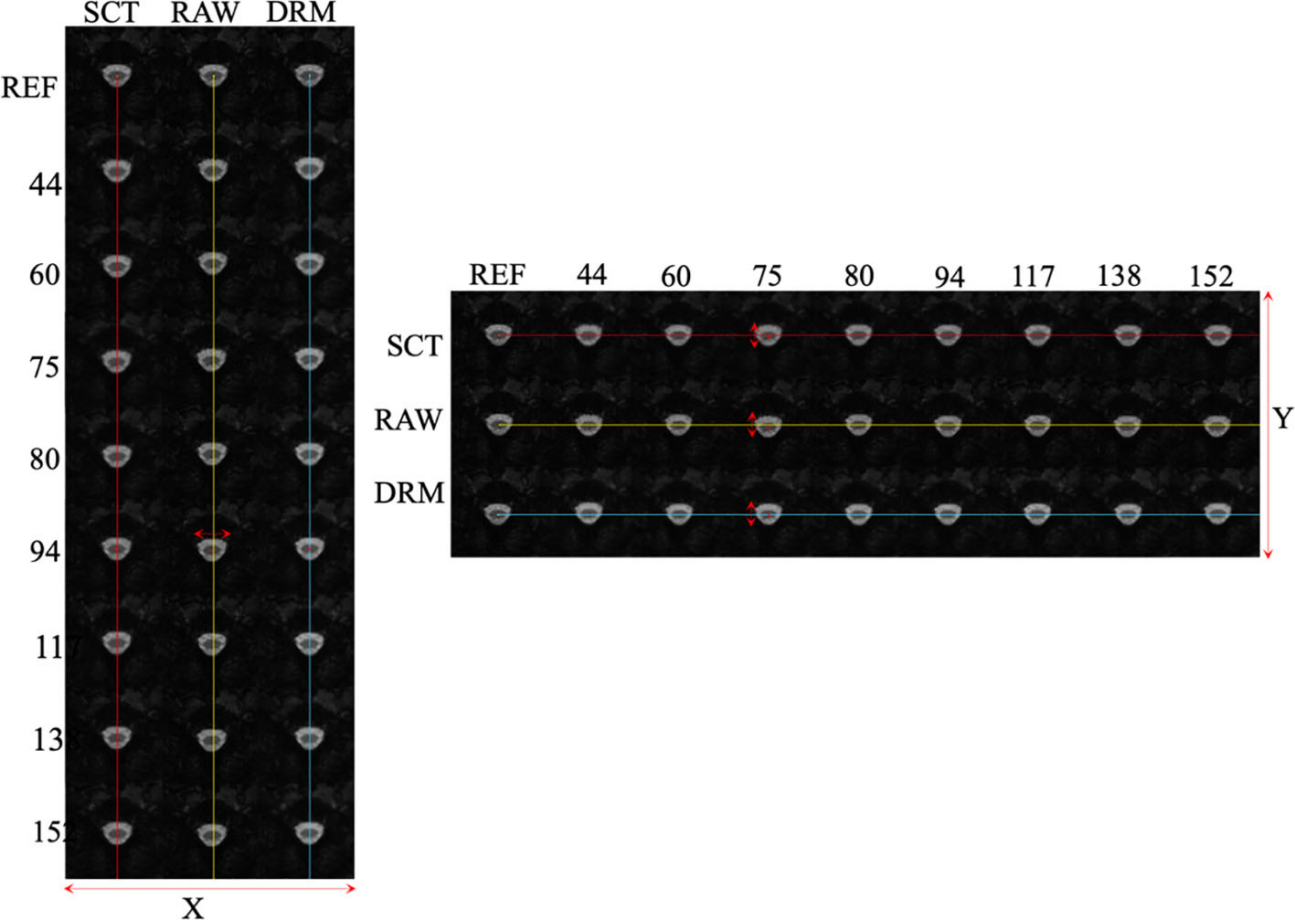
Comparative analysis of 3 protocols: SCT_fmri_moco (SCT), Deppretromoco (DRM), and raw data for Slice 4, showcasing precise centerline alignment across the x- and y-directions.

**Figure 4** presents a comparative evaluation of two motion correction techniques applied to fMRI data: sct_fmri_moco and Deepretromoco (DRM), juxtaposed with raw data. The results are demonstrated for a randomly selected subject at slice 4, with corrections displayed in two axes: the vertical (*x*-direction) and the horizontal (*y*-direction).

The center point on the reference volume, indicated on the figure, serves as the benchmark for evaluating the displacement of the centerlines across the volumes. The lines track the center points through subsequent volumes, highlighting the deviations from the reference.

In the vertical axis, the alignment of volume 94’s centerline with the reference illustrates the motion correction in the *x*-direction. The DRM method shows a fixed centerline, indicative of precise realignment, as opposed to the sct_fmri_moco method, where the centerline exhibits a discernible shift from the reference.

Conversely, in the horizontal axis, the alignment of the center points for volume 75 is examined. Here, the DRM method demonstrates a better match with the reference center point, suggesting a more accurate correction in the *y*-direction compared to the sct_fmri_moco method.

### 3.3 Statistical comparison of motion correction protocols

A one-way repeated-measures ANOVA was used to compare the influence of motion correction techniques on test data in sct_fmri_moco (12) and DeepRetroMoco, a deep neural network-based motion correction tool.

**Figure 5:**
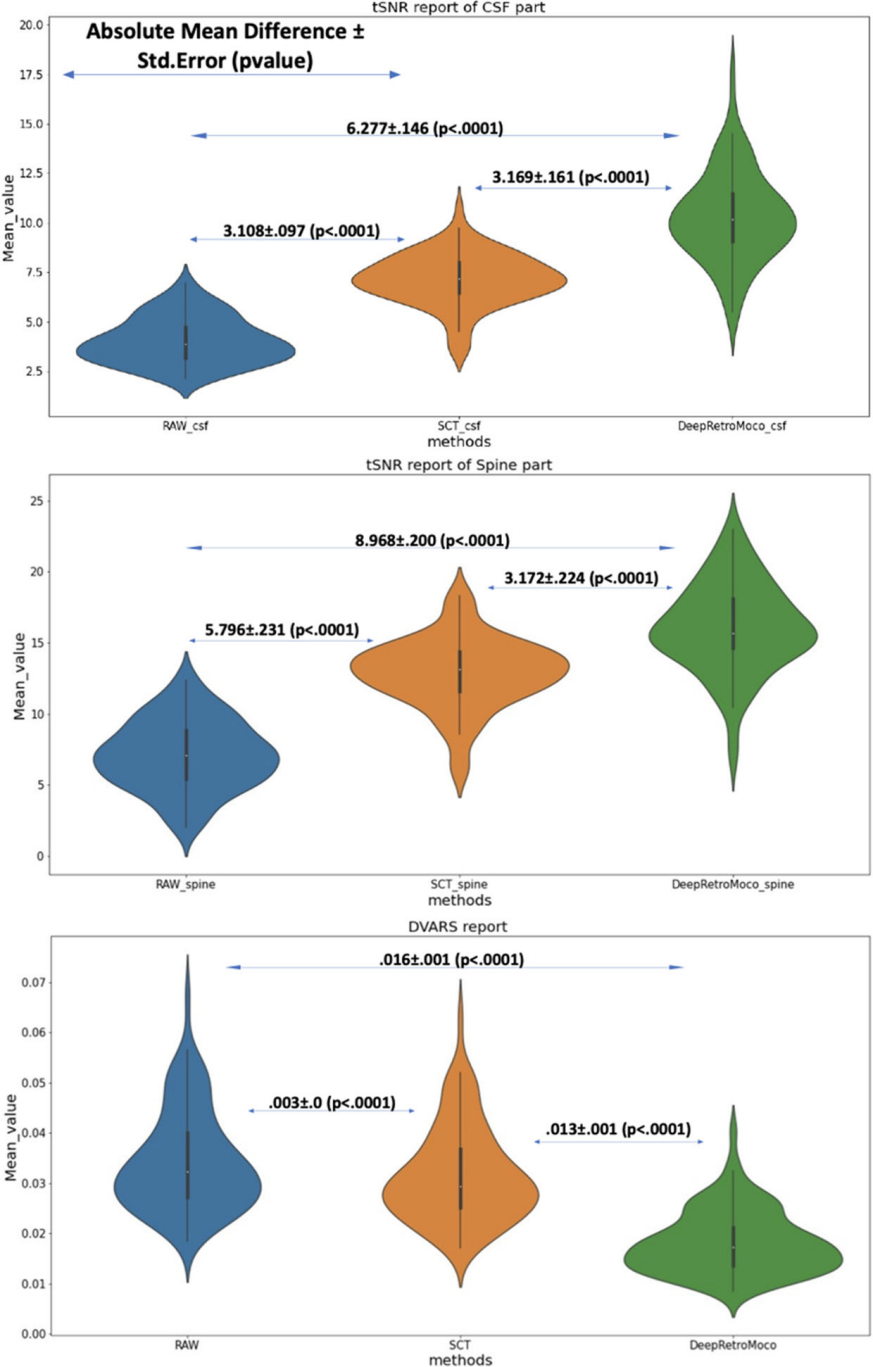
This figure depicts the mean and standard deviation of the SNR on the Spine and CSF sections (two top figures) that were manually segmented, as well as DVARS (bottom figure) with three types of results. RAW data that have not been corrected, SCT results, and DeepRetroMoco results are the three groups. The absolute mean difference + standard error (p-value) between groups is also reported. The mean difference is significant at the 0.05 level.

In a statistical comparison of tSNR parameters in the spinal cord, this parameter increased significantly from 7.104 ± 2.41 to 16.072 ± 3.09 arbitrary units (AU) (**Table 2**). Mauchly’s Test of Sphericity revealed that the assumption of sphericity had been violated, χ^2^(9) = 2.324, *p*< 0.313, and thus a Greenhouse–Geisser correction was used. The motion correction algorithm had a significant effect on the tSNR parameter in the spinal cord, *F*(2, 160) = 862.572, *p*< 0.0001. *Post-hoc* multiple comparisons using the Bonferroni correction revealed that the DeepRetroMoCo had a significantly higher mean tSNR in the spinal cord than the other motion correction method and raw data (*p*< 0.0001). **Figure 5** depicts the significant difference between the groups using a violin plot.

**Table 2:** Summary of tSNR as an image quality parameter between different motion correction methods (df = 4).

tSNR		Mean (SD)	*F*-value	*p*-value
Spinal cord	Raw image	7.1043 (2.41)	1,004.249	<0.001
	sct_fmri_moco	12.90 (2.44)		
	DeepRetroMoCo	16.072 (3.09)		
CSF	Raw Image	4.0387 (1.17)	938.842	<0.001
	sct_fmri_moco	7.1469 (1.31)		
	DeepRetroMoCo	10.3156 (2.25)		

The tSNR in CSF increased significantly from 4.038 ± 1.17 to 10.315 ± 2.25 AU (**Tables 2**, **3**). Mauchly’s Test of Sphericity revealed that the sphericity assumption had been violated, χ^2^(9) = 27.772, *p*< 0.0001, and thus a Greenhouse–Geisser correction was applied. The motion correction algorithm had a significant effect on the tSNR parameter in CSF, *F*(2, 160) = 949.72, *p*< 0.0001. *Post-hoc* multiple comparisons using the Bonferroni correction revealed that the DeepRetroMoCo’s mean tSNR in CSF was significantly higher than the other motion correction method and raw data (*p*< 0.0001) (**Table 2**). **Figure 5** depicts the significant difference between the groups using a violin plot.

**Table 3:** Average tSNR for two types of our model, DRM-1 and DRM-2. Standard deviations are in parentheses.

Model	Loss type	Mean tSNR (SD)	*F*-value	*p*-value
DRM_1	MSE	6.18(0.6)	12.408	<0.001
DRM_2		5.96 (0.7)		
DRM_1	NCC	10.13(1)	2.632	<0.05
DRM_2		10.07 (1.3)		

The averages are computed over all validation data. In both models, regardless of the type of cost function, the first model is selected (df = 39).

DVARS decreased statistically significantly from 0.034 ± 0.009 to 0.018± 0.006 AU (**Table 4**). Mauchly’s Test of Sphericity revealed that the sphericity assumption had been violated, χ^2^(9) = 64.966, *p*< 0.0001, and thus a Greenhouse–Geisser correction was applied. The motion correction algorithm had a significant effect on the DVARS parameter, *F*(2, 160) = 309.349, *p*< 0.0001. *Post-hoc* multiple comparisons using the Bonferroni correction revealed that the DeepRetroMoCo had significantly lower DVARS than the other motion correction methods and raw data (*p*< 0.0001) (**Table 4**). **Figure 5** depicts the significant difference between the groups using a violin plot.

**Table 4:** Summary of DVARS as an image quality parameter between different motion correction methods (df = 4).

DVARS	Mean (SD)	*F*-value	*p*-value
Raw image	0.0343 (0.009)	176.446	<0.001
sct_fmri_moco	0.0316 (0.009)		
DeepRetroMoCo	0.0182 (0.006)		

#### 3.3.1 Reference volume impact on motion correction

To elucidate the impact of different reference volumes on motion correction efficacy in spinal cord fMRI, our study systematically evaluates first, mid, and mean volume references. Our findings, as depicted in **Table 5**, aim to establish a guideline for selecting the most effective reference volume to maximize motion correction accuracy, enhancing spinal cord fMRI’s reliability for both research and clinical applications. It is noteworthy that while the first and mid-volume references were derived post-centerline alignment (the first stage of correction), the mean volume reference utilized was obtained before this alignment stage. This delineation underscores a significant area for methodological refinement. Employing the mean volume result from the initial correction stage as a reference for subsequent analyses presents a promising avenue for future research, potentially offering a more accurate basis for motion correction. This strategic adjustment could further improve motion correction outcomes, contributing to the precision and dependability of spinal cord fMRI analyses.

**Table 5:** Summary of tSNR and DVARS for spinal cord and CSF across different methods (post-correction*, SCT, and DRM) with varied reference volumes.

Methods		Spinal cord	CSF	Mean (SD)	
		Mean (SD)			
**tSNR**	Post-correction *	7.1 (2.41)	4.03 (1.17)	**DVARS**	0.03 (0.010)
	SCT_first-volume	12.9 (2.44)	7.14 (1.31)		0.03 (0.009)
	SCT_mean-volume	12.1 (2.52)	7 (1.15)		0.03 (0.009)
	DRM_first-volume	16.07 (3.09)	10.31 (2.25)		0.02 (0.011)
	DRM_mid-volume	13.65 (2.51)	9.06 (1.78)		0.02 (0.012)
	DRM_mean-volume	13.17 (2.99)	8.59 (2.3)		0.02 (0.010)

*Post-correction refers to the results after centerline alignment. See section 3.2.1 for more information.

### 3.4 Statistical comparison with other methods

In this study, we employed FSL’s MC_FLIRT for movement estimation across three groups of data: RAW (uncorrected), SCT toolbox results (sct_fmri_moco), and DeepRetroMoCo outcomes. The first volume served as the reference with a 6-degree of freedom setting for motion estimation. We analyzed the results using the MSE parameter, aligning actual movement to a zero baseline and comparing against movements predicted by FSL. **Table 6** shows the raw data demonstrating the most movement in all directions, followed by SCT and DeepRetroMoCo results. This approach allowed us to assess the effectiveness of our DeepRetroMoCo method in comparison to the established methods.

**Table 6:** Mean square error of three groups of our data in six directions such as translation in X, Y, and Z and rotation in X-, Y-, and Z-directions.

MSE	Translation (mm)			Rotation (radian)		
Dir	*X*	*Y*	*Z*	RX	RY	RZ
**DeepRertoMoco**	1.41E-07	2.06E-07	5.43E-07	0.001	0.0001	0.0023
**SCT**	5.54E-06	3.91E-07	1.47E-06	0.0012	0.0103	0.0054
**RAW data**	5.61E-06	1.15E-05	1.55E-06	0.1435	0.1073	0.0017

### 3.5 Processing speed

**Figure 6:**
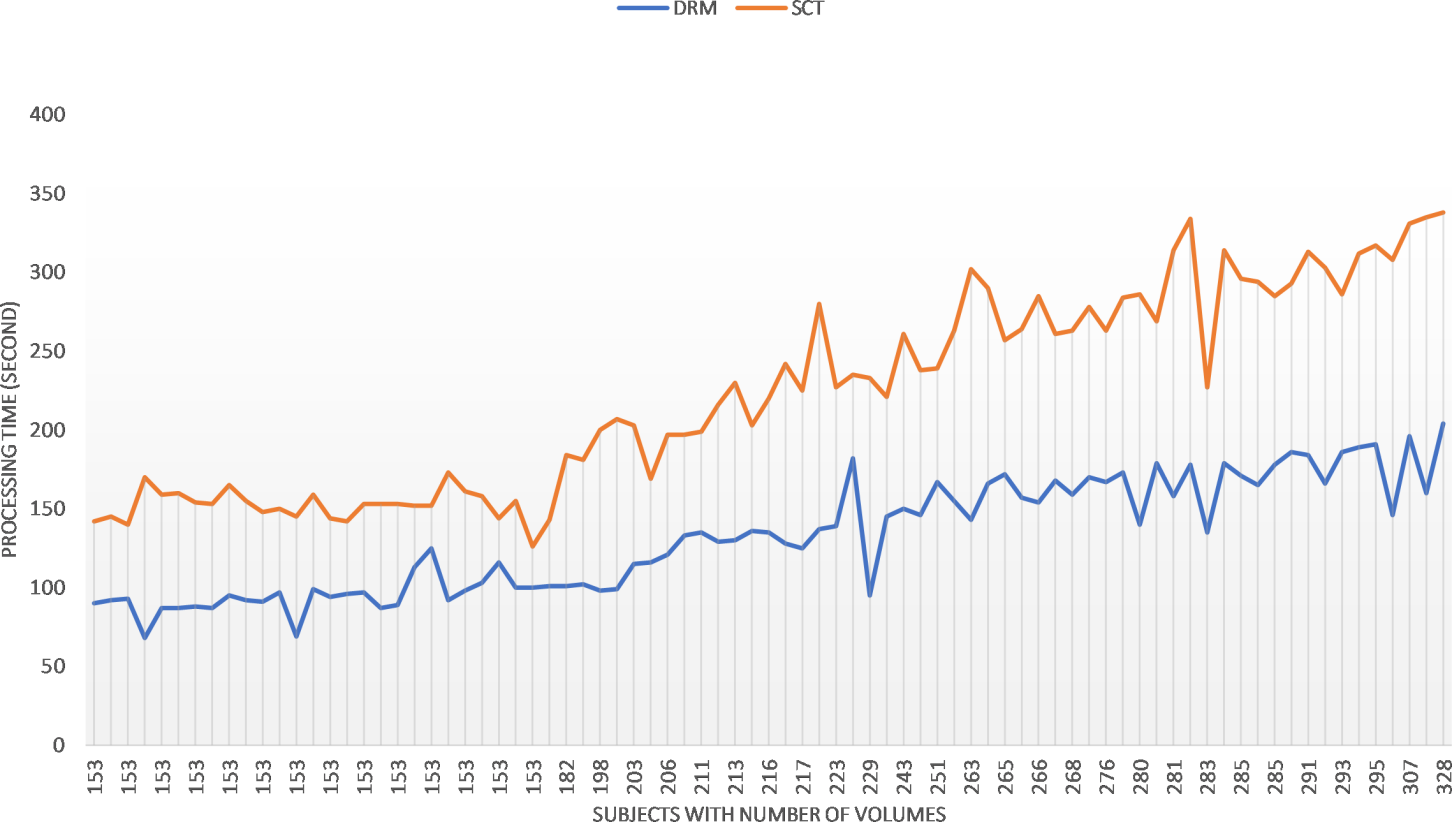
Comparing the speed of the two methods sct_fmri_moco (SCT) and DeepRetroMoco (DRM). Processing time is measured in seconds to correct the motion on all volumes.

The implementation and calculation are carried out in a workstation with Intel^®^ Core (TM) i7–4720HQ CPU at 2.60 Hz and 16.0 GB memory. No explicit parallelization was implemented in the Python script. The computation time of the motion correction procedure in sct_fmri_moco and DeepRetroMoco changes with the number of volumes of fMRI raw data (**Figure 6**). Average computation times (± SD) were 222.54 ± 63.64 s and 131.91 ± 35.94 s for sct_fmri_moco and DeepRetroMoco respectively and demonstrates a significant reduction of ~40.72% in computation time. This operation for SCT contains the slice-by-slice registration plus regularization across the Z, and that for DeepRetroMoCo contains fixing the centerline plus registration via a network.

### 3.6 Regularization analysis

**Figure 7:**
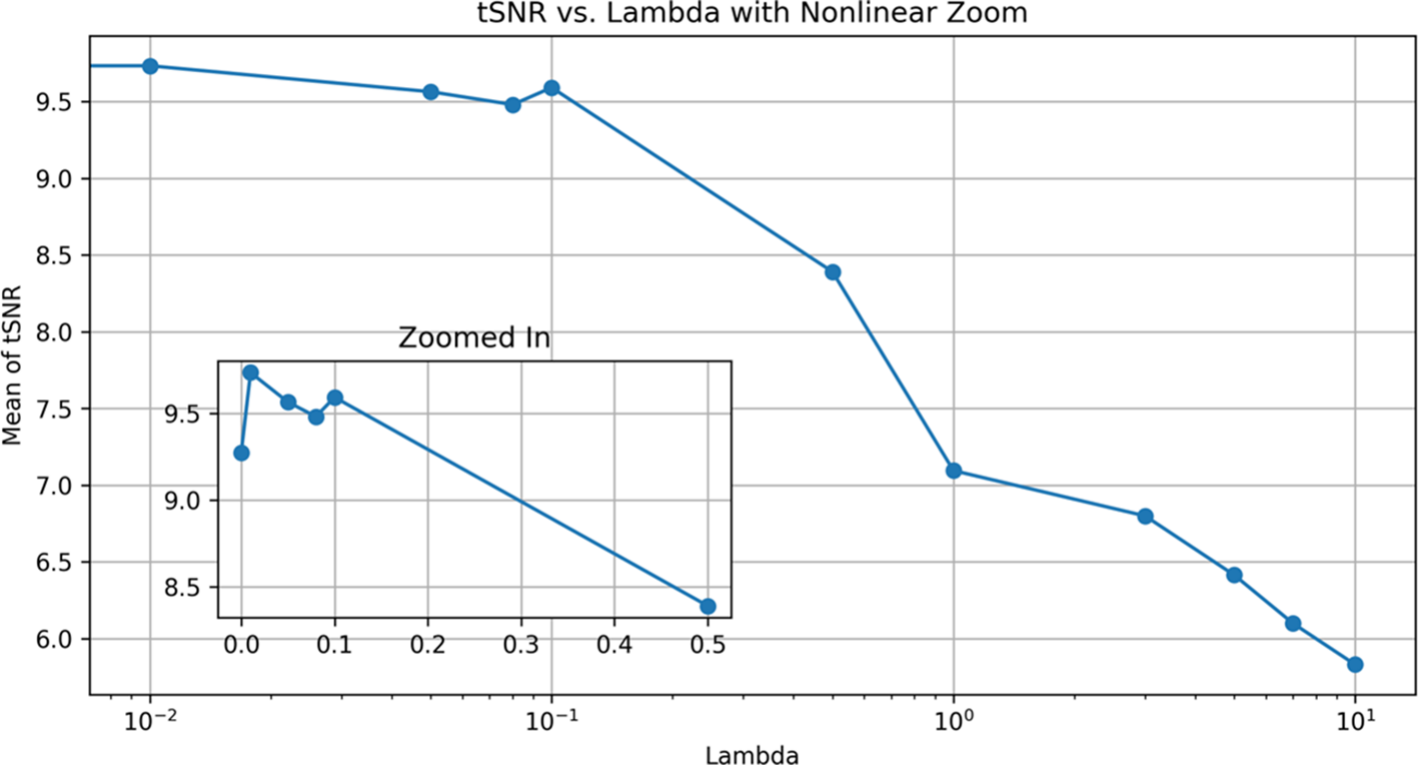
Effect of different λ modes for DRM_1 based on tSNR. Lambda 0.01 has a maximum tSNR and shows the best results.

With different lambda parameters, we examined the mean tSNR for the test data. With the NCC cost function, the optimal tSNR for model 1 occurred when lambda was 0.01. In this section, the mean tSNR is applied to the entire spinal cord; lambda = 0 indicates no regularization. As shown, the results deteriorate dramatically as the regularization term is increased (**Figure 7**). As a result, lambda’s actions do not help to improve performance and may have a negative impact on the results for the NCC cost function and the first model, which is more complex.

### 3.7 Correlation coefficient analysis

In our comparative analysis, we also evaluated the performance of SCT’s sct_fmri_moco method by calculating the Pearson correlation coefficient (CC) between the corrected and reference volumes. The CC value for SCT’s sct_fmri_moco was observed to be 0.82 ± 0.03, which, while indicating an improvement over the raw data (0.70 ± 0.17), is notably lower than the CC value achieved with DeepRetroMoCo (0.90 ± 0.02). This comparative assessment further highlights the superior performance of DeepRetroMoCo in enhancing the linear similarity of the images post-correction, demonstrating its effectiveness in motion correction while preserving the integrity of the original image structure. The inclusion of SCT’s sct_fmri_moco in our analysis provides a comprehensive perspective on the advancements our method offers over existing techniques in the domain of spinal cord fMRI data correction.

## 4 Discussion

Since the spinal column’s voluntary and non-voluntary movements lead to non-optimal shimming, the effects of motion artifacts cannot be fully eliminated even after perfect conventional retrospective motion correction of successive functional volumes in the image space (47). If spinal column movements are small, motion correction is a useful step to improve the data quality for subsequent statistical data analysis. Our findings demonstrate that deep learning-based motion correction, DeepRetroMoco, improves the quality of spinal cord fMRI data acquired in the axial field of view that influences the pre-processing step. These improvements are at least in part due to improved tSNR and DVARS parameters compared to conventional algorithms introduced in the SCT data processing toolbox. Instead, here we aimed to use a deep learning-based method potential to decrease the preprocessing step for spinal cord fMRI data strongly affected by motion. We found significant differences in the time of processing to implement DeepRetroMoco compared to the sct_fmri_moco algorithm.

As previously mentioned, the majority of leaning-based methodologies require additional data or ground truth. We do not need this information, which is another clear distinction between our approach and earlier research. The previous two works (48, 49) reported unsupervised methods that are close to ours. Both use the CNN neural network with STF (39), which warps images on top of each other and has significant problems: they only operate on a limited subset of volumes and only support small transformations. In addition, a recent study (50) and our network improved the problems mentioned and helped to solve them by designing a satisfactory model in the spinal cord data. Other methods (49) use regularization that is determined only by interpolation methods.

DeepRetroMoco replaces a costly optimization problem for each image pair, with a function optimization that is collected over a dataset during a training step. This notion could be replaced with previous motion correction algorithms, especially on spinal cord data that traditionally rely on complex, non-learning-based optimization algorithms for each input. Although implementing this network requires a one-time network training on a single NVIDIA TITAN X GPU with training data, it takes less than a second to register a pair of images. Because of the growing need for medical images for further investigation in less time, our solution, which is a learning-based method, is preferable to non-learning-based methods.

Our DeepRetroMoCo method’s effectiveness is partly due to the initial centerline alignment preprocessing. Initially, the model without preprocessing showed limited improvement in motion artifact correction. Integrating the centerline alignment step marked a significant enhancement, facilitating more effective motion correction, particularly in the key directions of spinal movement. This preprocessing step, in conjunction with the neural network’s capabilities, forms a cohesive strategy, significantly improving motion correction efficacy as demonstrated by our improved tSNR and DVARS metrics.

### 4.1 Limitations and future works

The acquisition of spinal cord fMRI data is made in two ways: GRE-EPI acquisition sequence in axial and FSE or SE-HASTE acquisition sequence in sagittal field of view. The field of view and dataset orientation were axial in this study, and all motion correction methods and preprocessing steps were performed specifically on axially oriented data in the cervical spine; however, some studies performed spinal cord fMRI acquisition in the sagittal orientation.

Furthermore, we had access to two variables during this method: the centerline reference and the fixed image reference. It was set to the first volume in our network. We discovered that the proper selection of these two parameters could have a significant impact on the final results. Because our network is flexible enough to accept any reference, including first, mean, middle, and any other desired volume, we propose that the best reference for each data be selected by designing the appropriate method for future work.

An additional limitation to consider is the effect of B0 field fluctuations on the apparent translational motion in spinal cord EPI images. Our DeepRetroMoCo method, in its current state, does not explicitly differentiate between motion artifacts stemming from subject movement and those induced by temporal fluctuations in the B0 field. This distinction is particularly relevant because B0 fluctuations can significantly affect GRE-EPI images, which is the acquisition sequence used in our study. In future iterations of our research, we intend to address this limitation by integrating B0 field map information into the DeepRetroMoCo framework to enhance its capability to accurately correct for these specific types of artifacts.

While our study provides a solid foundation for the application of DeepRetroMoCo in spinal cord fMRI data processing, it is important to acknowledge that the method was trained and tested on a single, highly homogeneous dataset. This approach was chosen to initially establish the method’s efficacy under controlled conditions. Moving forward, our research aims to evaluate the performance of DeepRetroMoCo across additional, more varied datasets. This expansion is crucial for assessing the method’s generalizability and robustness to different imaging characteristics and to ensure its applicability in broader clinical settings. Furthermore, incorporating datasets not used in the current study will allow us to test the method’s adaptability and fine-tune its parameters for a wider range of applications. This future work will be pivotal in determining the full potential of DeepRetroMoCo for widespread clinical use and will contribute significantly to its development to meet the diverse needs of spinal cord imaging research.

Our observations also highlighted the presence of ghosting effects, particularly in slices closer to the lungs, where respiratory motion significantly impacts image quality. Such artifacts, driven by a combination of respiratory and cardiac motion, patient movement, field inhomogeneities, and phase encoding artifacts, underscore the complexity of spinal cord fMRI data acquisition. Despite the robust motion correction capabilities of DeepRetroMoCo, slices exhibiting pronounced ghosting effects due to these factors presented a challenge, with a slight decrease in performance observed in terms of tSNR. This underlines the inherent difficulty in completely eliminating motion artifacts, especially in areas with severe geometric distortions or near intervertebral discs where shimming is suboptimal. These findings further emphasize the need for sophisticated motion correction strategies that are sensitive to the unique challenges presented by spinal cord fMRI data.

## 5 Conclusion

Owing to the bulk and physiological motion corrupted spinal cord fMRI data, the statistical significance of the activation maps decreases, and the likelihood of false activations increases. As a result, a motion correction algorithm is required for acceptable single and group fMRI data analysis. In this study, we proposed DeepRetroMoco, an unsupervised learning-based approach based on advanced CNN models, which requires no supervised information such as ground truth registration fields or anatomical landmarks. Additionally, when compared to conventional methods, the use of the DeepRetroMoco motion correction method for spinal cord fMRI shows remarkable effectiveness in enhancing tSNR, decreasing false positives, and improving sensitivity, particularly in scenarios involving the substantial motion of the spinal cord. Additionally, our evaluation of DVARS as an fMRI quality metric, along with its timely implementation on a cervical spinal cord fMRI dataset, underscores the superiority of our proposed framework in our experimental investigation. Moreover, this method serves as a straightforward and seamless tool for achieving more precise and efficient motion correction for denoising purposes in spinal cord fMRI applications.

## Data availability statement

The original contributions presented in the study are included in the article/supplementary material, further inquiries can be directed to JD (julien.doyon@mcgill.ca) or the corresponding author.

## Ethics statement

The studies involving humans were approved by Committee at the Centre de Recherche de l’Institut Universitaire de Gériatrie de Montréal (CRIUGM). The studies were conducted in accordance with the local legislation and institutional requirements. The participants provided their written informed consent to participate in this study.

## Author contributions

MM-A: Writing – review & editing, Writing – original draft, Methodology, Investigation, Formal analysis, Data curation. AM-A: Writing – review & editing, Validation, Supervision, Software, Methodology, Conceptualization. HD: Writing – review & editing, Investigation, Formal analysis, Data curation. MZ: Writing – review & editing, Supervision. SV: Writing – review & editing, Writing – original draft, Supervision, Data curation. JD: Writing – review & editing, Supervision, Resources. AK: Writing – review & editing, Writing – original draft, Visualization, Validation, Supervision, Resources, Project administration, Methodology, Investigation, Formal analysis, Data curation, Conceptualization.
